# How live stream content types impact viewers’ support behaviors? Mediational analysis on psychological and social gratifications

**DOI:** 10.3389/fpsyg.2022.951055

**Published:** 2022-10-11

**Authors:** Eric Mao

**Affiliations:** School of Cultural Creativity and Management, Communication University of Zhejiang, Hangzhou, China

**Keywords:** live streaming, uses and gratifications, content type, support behavior, new media

## Abstract

While previous research into live streaming was predominantly focused on video games, its content creation and provision has tremendously evolved, extending well-beyond game streams. Contents of general interest, such as e-commerce shopping, talent shows, and cute pets, started to prevail in today’s landscape of live streaming. However, limited attention has been given to how distinct types of streaming contents influence viewers’ psychological and behavioral responses. To fill this void, we employed an online survey (*n* = 583) to empirically examine the associations between popular live stream content types on *Douyin* (i.e., the *TikTok* app for China) and their viewers’ psychological and social gratifications and typical support behaviors. The results revealed that gratifications varied drastically across different content types. Game streaming, in particular, generated significant indirect impacts on all the support behaviors under consideration. On the other hand, whereas tension release served as a consistent mediator, the cognitive needs had no significant mediation effects. In sum, our study makes theoretical contributions to the literature by analyzing the thriving live stream phenomenon from a uses and gratifications perspective. We help augment the understanding of new media users’ preferences and choices in an attention economy, wherein human attention is conceptualized as a scarce resource. In practice, a better knowledge of viewer needs can facilitate streamers to customize their content creation and provision so as to accentuate elements of interest and elicit desired support behaviors (i.e., monetization opportunities).

## Introduction

Live streaming is an interactive form of online entertainment activity, which has been gaining traction since early 2010’s (Hilvert-Bruce et al., 2018). According to a couple of market reports, in 2020 there were over 700 million live stream audiences across the world (Newzoo, 2021), while its global market size was projected to reach US$50.11 billion (Grand View Research, 2021). At its early stage, the live stream ecosystem was primarily dominated by video gaming contents like competitive esports tournaments, instructions on gameplay strategies and intricacies, and casual play without definite goals (Sjöblom et al., 2017). However, the recent landscape of live streaming has been greatly reshaped by an enriched variety of content provision. For example, a market survey conducted in May 2020 revealed that shopping, food, gaming, and beauty-oriented contents accounted for the leading types of live streaming in China (Statista, 2020). Today, it is not uncommon to see streams broadcasting talent shows performed by celebrities or amateurs, outdoor recreation taking place in natural or urban settings, pet feeding spotlighting domestic or wild animals, as well as other interesting programs. The rapid proliferation and diversification of live stream contents is said to be inspired by “The democratization process of content creation” (Sjöblom et al., 2017, p. 161), where small entities and individuals outperform large corporations and organizations in providing original and customized audiovisual media products.

In response to the highly diligent and productive streaming content suppliers, there are a multitude of avenues for viewers to support their recognized channels. Depending on the amount of effort and money entailed, the support behaviors can be stratified into different levels. To begin with, as an effortless and costless option of embracing the channel, “continuous watching” can help enlarge the number of concurrent viewers and add to the volume of Internet traffic (Hou et al., 2019; Lv et al., 2022). Nothing needs to be done but giving attention to the streams continuously or intermittently. Second, tapping the “like” and “follow” (or “subscribe”) buttons is free of charge and merely requires a minimum amount of effort (Recktenwald, 2017). The difference resides in the sense that, whereas the former can be interpreted as viewers’ one-off approval of the current content, the latter signifies a strong willingness to regularly receive channel-specific updates in the future, or put it differently, to establish relational bonds with the channel/streamer as committed followers. Live stream viewers can choose to execute either one of them independently, or both at once. Third, to “share” the on-going live streaming is realized by sending private messages to online friends or forwarding it to other social media platforms (Martini, 2018; Wongkitrungrueng et al., 2020). Adopting the “share” action implies that viewers voluntarily engage in disseminating the content of interest, who usually do so for the sake of seeking emotional resonance. Fourth, making real-time “comment” or sending “danmaku” represents a higher level of support as viewers deliberately express their personal thoughts and feelings related or unrelated to the streamed content, in hope of launching social interactions with the streamer(s) as well as other ones in the channel (Haimson and Tang, 2017; Luo et al., 2020; Li and Guo, 2021). Finally, “tipping” can be thought of as the highest level of support within the context of live streaming, mainly in the form of gratified viewers buying and giving virtual gifts reconvertible to real money (Lin et al., 2021; Lu et al., 2021). Income from “tipping” or “donation” often constitutes a significant source of financial compensation for providing streaming services. It should be noted, however, that the above-mentioned hierarchical order is not necessarily clear-cut considering the support behaviors in question may have differing focuses that are not directly rankable based on certain criteria.

Existing communication and e-commerce literature has inspected the phenomenon of live streaming through the lens of the uses and gratifications (U&G) theory. Given a considerable body of research was predominantly focused on viewing motivations (Hilvert-Bruce et al., 2018; Zimmer et al., 2018; Cai and Wohn, 2019; Steiner and Xu, 2020), there were also inquiries into live stream viewers’ loyalty (Hsu et al., 2020), shopping intentions (Ma, 2021), and gift giving (Li and Guo, 2021). Furthermore, recent research suggested that live streaming can be utilized for many specialized services, such as telehealth and telemedicine (Arunkumar et al., 2019), agricultural data visualization (Kannimuthu et al., 2019), and pattern extraction for web service (Kannimuthu et al., 2012; Kannimuthu and Chakravarthy, 2022). However, to our best knowledge, few studies have investigated how different content types of live streaming are associated with viewers’ specific needs, and once these needs are satisfied, to what extent or in what form viewers would support their favorite channels/streamers. Considering the information explosion in today’s society, it is warranted to expand our understanding of varied live stream content types because human attention becomes increasingly scarce compared to the overflow of information (Davenport and Beck, 2001) and we are only capable of consuming a narrow array of media content. In turn, a better knowledge of viewer needs can facilitate streamers to customize their content creation and provision so as to accentuate elements of interest and elicit desired support behaviors, namely monetization opportunities. As such, to fill the research void, the current study conducted an online survey to collect information about users’ frequencies of consuming live stream contents with distinct themes on *Douyin*, i.e., the *TikTok* app for users in mainland China, and then measured their psychological and social gratifications in addition to the behavioral tendencies to support the channel/streamer. Structural equation modeling (SEM) analysis, with the inclusion of mediation effects, was employed to statistically anatomize a sample of 583 self-identified active users of *Douyin*’s live stream services.

## Backgrounds and research questions

While there exist multiple platforms that have live stream sections and can be used for research purposes, such as *Twitch*, *Facebook*, and *Taobao*, our study focused solely on *Douyin*. The reasons are 2-fold: (1) *Douyin*’s daily active users (DAU) had surpassed 600 million as of August 2020 (Choudhury, 2020); and (2) Compared to other platforms, which are generally characterized by single-themed content like gaming or shopping, *Douyin*’s live stream ecosystem is much more comprehensive in that more than 25 types of contents are affluent and easily accessible. According to information compiled from several data analysis and visualization websites specialized in *Douyin*, including *Ocean Insights*,^1^ *Chanmama*,^2^ and *Feigua*,^3^ the top ten most popular^4^ types of live streaming on *Douyin* contain e-commerce shopping, beauty influencers, talent shows, entertaining plots, video games, food, education, sports and fitness, cute pets, and cosmetics. Table 1 provides further details about these content types. Notably, some otherwise intuitively popular live stream themes like fashion, tourism, and product tutorial and review cannot be on the list because they are comparatively less attention-grabbing. Thus, we only used the top ten content types of *Douyin* live streaming in the analysis.

**TABLE 1:** Live stream content types and descriptions.

Content type	Description
E-commerce shopping	Celebrities, Internet influencers, or amateur salespeople selling branded or unbranded products.
Beauty influencers	Individuals with good-looking appearance, usually in the use of beautifying filters.
Talent shows	Exhibition of personal talents and skills such as singing, dancing, handcrafting, painting, and other creative abilities.
Entertaining plots	Short episodes or scenes with disproportionate emphasis on funny and humorous elements.
Video games	Esports competitions, introductions to newly released games, female gamers’ play, and etc.
Food	Mukbang, restaurant review, and cooking instructions.
Education	Knowledge sharing and training courses on varied topics like healthcare and financial investment.
Sports and fitness	Broadcasting of sports contests and other events; Professional fitness trainers demonstrating how to work out in real-time.
Cute pets	Mostly cute cats and dogs, domestic or wild, as well as other relatively less common pets, such as hamsters and lizards.
Cosmetics	Beauty experts showing how to select decent and affordable cosmetic products and how to apply and remove makeup.

Regarding the theoretical background, an audience-centric approach was applied to deepen the understanding of the relationships between live stream viewers’ preferred content types, specific needs and satisfaction related to media consumption, and the resultant support behaviors. That is, the well-known U&G theory elucidates why and how users proactively seek out and consume certain media and media contents in order to satisfy particular social and psychological needs (Katz et al., 1973a,b; West and Turner, 2009). It affords a framework for justifying the role of users’ preferences and choices in media engagement as a consequence of fulfilling idiosyncratic needs (Hilvert-Bruce et al., 2018). Having served as a robust theoretical underpinning, the U&G framework is often utilized to explain various media usages, including television and radio (Rubin, 1983; Albarran et al., 2007; Papacharissi and Mendelson, 2007), social media (Whiting and Williams, 2013; Dolan et al., 2016; Quinn, 2016), video games (Wu et al., 2010; Puri and Pugliese, 2012; Hamari et al., 2019), and virtual reality (Kim et al., 2020; Ball et al., 2021; Kim and Lee, 2022).

It is well-established in the previous U&G research that media consumers are typically motivated by five distinctive types of needs: cognitive, affective, personal integrative, social integrative, and tension release (Sangwan, 2005; Sjöblom and Hamari, 2017; Sjöblom et al., 2017; Can et al., 2019). In particular, within the context of live streaming, information seeking (Hilvert-Bruce et al., 2018; Chen and Chang, 2019) and learning (Payne et al., 2017) have been identified as two ubiquitous cognitive motivations. The affective motivation revolves around a sense of enjoyment, derived from watching the streams *per se* (Wulf et al., 2020; Singh et al., 2021) or from interacting with peers in the channel (Cai and Wohn, 2019). The personal integrative motivation is often referred to as the self-esteem need, with a focus on reassuring media viewers’ status and gaining credibility, whereas the social integrative motivation encompasses the intrinsic need to socialize with family, friends, as well as other relations in the society (Sjöblom and Hamari, 2017). Finally, the tension release motivation pertains largely to escapism and diversion from daily routines and/or life difficulties (Sangwan, 2005).

**FIGURE 1:**
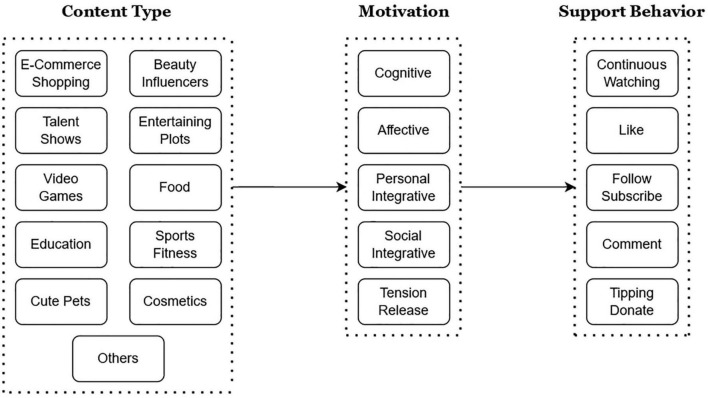
Conceptual research model.

The above-mentioned motivations for media use are closely aligned with different types of live streaming. For example, it is natural for individuals who have information seeking and learning needs to be more likely to watch news and education contents. In a similar vein, live stream viewers focused on affective values should inherently prefer entertaining genres, such as singing and dancing, and those who are in quest of personal integrative values can have an increased chance to join a shopping channel to browse and buy luxury goods, as a means of maintaining self-confidence. On the other hand, the relevant literature has broadly discussed media users’ behavioral responses when their specific needs are met. In Sjöblom and Hamari’s (2017) work, it was found that the affective, social integrative, and tension release gratifications positively predicted how much time was spent on watching video game live streaming. They also found that viewers’ propensity to subscribe became stronger if their social integrative needs were satisfied. Likewise, using minute-level live stream data, Lin et al. (2021) statistically analyzed the dynamics between the real-time sentiments of the broadcaster and viewers, and the resultant effects on the latter’s support behaviors including “like,” “comment,” and “tipping.” Interestingly, their results indicated that live streamers’ positive emotions can be contagious to viewers in the same channel, which in turn boosted the number of likes and the amount of tipping. With these in mind, we propose a conceptual research model (see Figure 1) and the following research questions for the current study:

RQ1. How are psychological and social gratifications derived from consuming live stream service associated with content types?

RQ2. How are live stream viewers’ support behaviors associated with psychological and social gratifications?

RQ3. Do psychological and social gratifications mediate the relationships between live streams’ content types and viewers’ support behaviors?

## Materials and methods

### Procedure and sampling

A pilot study with 30 respondents was conducted prior to launching the primary survey to check whether there existed potential issues in the questionnaire design. After no major problems were detected, an invitation link to the survey, powered by *Tencent Questionnaire*, was disseminated *via* the social media app *WeChat* to college students of three universities in East China (i.e., cluster sampling). It roughly took a month for finishing the data collection process, from mid-April to mid-May 2022. In total, 596 responses were successfully gleaned, with 13 (2.2%) samples being eliminated owing to invalid answers (e.g., 180 for age) or repetitive patterns in the answers (e.g., one for all the items), ultimately resulting in 583 valid responses. 63.46% of the respondents were female (*n* = 370), and their average age was 20.47 (SD = 2.31).

### Construct measurement

A five-point frequency scale (“never,” “seldom,” “sometimes,” “often,” and “always”), in which one indicates “never” and five for “always,” was capitalized on to measure how often the respondent watched the live stream content types under consideration. Second, the psychological and social gratifications were evaluated using the psychometric items previously adopted in two related papers authored by Sjöblom and Hamari (2017) and Sjöblom et al. (2017). Although their works were focused particularly on video game live streaming on Twitch, the items can be naturally applied to streams of more general interest, with only minor modifications being required. In addition, the scales developed by Papacharissi and Rubin (2000) and van der Heijden (2004) were merged to measure the respondent’s information seeking and learning (i.e., cognitive) motivations. Scales were only slightly different in terms of the contextual information, for example, “Twitch” was replaced with “live streaming” and a couple of repetitive items were removed. All the items in relation to the psychological and social motivations were graded on a seven-point Likert scale, with one denoting “strongly disagree” and seven denoting “strongly agree.” More details including the survey items, internal consistency evaluations, and summary statistics are displayed in Table 2. Lastly, another identical five-point frequency scale was used to measure how frequently the respondent was engaged in the aforementioned support behaviors, ranging from “never” to “always.” All the psychometric measures should be considered internally consistent provided the smallest value of Cronbach’s α was 0.88 (Table 2).

**TABLE 2:** Construct measurement and summary statistics.

Item	References	Cronbach’s α	Mean	SD
**Information seeking and learning (cognitive)**	Papacharissi and Rubin, 2000; van der Heijden, 2004	0.95		
ISL1: Watching live streaming, I make better informed decisions than in the past			3.44	1.75
ISL2: Watching live streaming, I am better informed about what is going on			4.08	1.70
ISL3: Watching live streaming, I find information I would not otherwise have found			4.29	1.78
ISL4: Watching live streaming, I am better informed about new knowledge			4.04	1.81
ISL5: Watching live streaming helps me look for information on new knowledge			4.28	1.83
ISL6: Watching live streaming helps me see what new knowledge is out there			4.46	1.76
**Perceived enjoyment (affective)**	Venkatesh, 2000	0.92		
ENJ1: I find watching live streaming to be enjoyable			3.68	1.74
ENJ2: Watching live streaming is exciting			3.32	1.79
ENJ3: I have fun watching live streaming			3.74	1.77
**Recognition by peers (personal integrative)**	Hernandez et al., 2011	0.93		
REC1: I like when other viewers take my comments into account			4.14	1.97
REC2: I feel good when my comments prove to other viewers that I have knowledge about the topics under discussion			4.44	1.97
REC3: I try that my comments improve my reputation among other viewers			3.54	1.98
REC4: I like when streamers take my suggestions			4.12	2.08
**Companionship (social integrative)**	Smock et al., 2011	0.88		
COM1: Watching live streaming, I don’t have to be alone			3.81	1.92
COM2: I watch live streaming when there’s no one else to talk or be with			3.16	2.03
COM3: Watching live streaming makes me feel less lonely			3.18	1.89
**Shared emotional connection (social integrative)**	Chavis et al., 2008	0.93		
SEC1: It is very important to me to be a part of the live stream community			2.33	1.70
SEC2: I spend time with other live stream community members a lot and enjoy spending time with them			2.15	1.55
SEC3: I expect to be a part of the live stream community for a long time			2.58	1.90
SEC4: Members of the live stream community have shared important events together			3.00	1.80
SEC5: Members of the live stream community care about each other			2.85	1.83
**Escape (tension release)**	Smock et al., 2011	0.91		
ESC1: Watching live streaming, I can forget about school, work, or other things			3.24	1.89
ESC2: Watching live streaming, I can get away from the rest of my family or others			3.01	1.85
ESC3: Watching live streaming, I can get away from what I’m doing			3.09	1.83
**Distraction (tension release)**	Smock et al., 2011	0.91		
DIS1: Watching live streaming is a habit, just something I do			2.62	1.80
DIS2: When I have nothing better to do, I watch live streaming			2.85	1.84
DIS3: Watching live streaming passes the time away, particularly when I’m bored			3.43	1.94
DIS4: Watching live streaming gives me something to do to occupy my time			2.81	1.77
**Relaxation (tension release)**	Smock et al., 2011	0.95		
REL1: Watching live streaming relaxes me			3.72	1.89
REL2: Watching live streaming allows me to unwind			3.60	1.88
REL3: Watching live streaming is a pleasant rest			3.74	1.94

### Statistical analysis

The package *lavaan* in the software R was used to conduct statistical analysis. First, we assessed how the psychological and social gratifications, including cognitive, affective, personal and social integrative, and tension release, were associated with the frequency of engaging in a specific type of live stream content (RQ1). We then tested whether the support-related behavioral tendencies increased if a certain need was satisfied (RQ2). Lastly, based on the results attained in the previous two steps, mediational analysis was undertaken to examine the mediation effects of the five psychological and social motivations (RQ3).

## Results and discussion

### Content types and gratifications

Table 3 reports the results concerning the relationships between live stream content types and viewers’ gratifications, from which it can be seen that distinct types of content differed drastically in fulfilling psychological and social needs. First, e-commerce shopping live streaming was observed to be positively associated with affective and personal integrative motivations. As a pioneer in the field of leveraging live streaming coupled with Internet influencers, China’s live commerce (or also known as live stream e-commerce) industry is typified by a wealth of humorous and dramatic elements (Meng et al., 2021; Guo et al., 2022), producing rich entertaining experiences for viewers. Meanwhile, a number of design features of the streaming channel, such as live Q&A with the host and comments that can be liked by others, substantially facilitate viewers to gain personal recognition and enhance their engagement. Such scenarios highly resonate with the literature of electronic word of mouth (eWOM) and social commerce, wherein a sense of recognition serves as a pivotal participation motive (Hennig-Thurau et al., 2004; Liang and Turban, 2011). Second, live stream viewers’ affective and social integrative gratifications increased when they were watching beauty influencers. Prior studies have well-documented the hedonic value derived from esthetic appreciation, especially when human faces and bodies are involved (Kreutzer and Aebischer, 2015; Swami et al., 2018). Moreover, search costs can be economized and sense of loneliness can be mitigated if it is easy to find discussants, with whom live stream viewers are able to share thoughts and feelings about a good-looking streamer, which explains why people happen to favor the most popular stars from an economic perspective (Adler, 1985).

**TABLE 3:** Direct effects of content types on gratifications.

Content type	Cognitive	Affective	Personal integrative	Social integrative	Tension release
E-commerce shopping	–0.002	0.349***	0.189*	0.053	0.062
Beauty influencers	0.050	0.183**	0.167	0.161**	0.091
Talent shows	–0.023	0.233**	–0.031	0.316***	0.171**
Entertaining plots	–0.031	0.151*	0.047	0.181**	0.163**
Video games	0.144***	0.317***	0.274***	0.316***	0.374***
Food	0.050	0.084	0.182*	0.160**	0.117*
Education	0.276***	–0.119	0.061	0.032	0.051
Sports and fitness	–0.129	–0.072	–0.099	–0.006	–0.105
Cute pets	0.011	0.173**	0.288***	–0.030	–0.020
Cosmetics	0.170**	–0.004	–0.036	–0.005	0.045
Others	0.005	–0.100	−0.182*	0.227***	0.127*
*R* ^2^	0.134	0.421	0.186	0.455	0.410

*p < 0.05, **p < 0.01, ***p < 0.001.

Third, similar to beauty influencer content, streams highlighting talent shows and entertaining plots were effective in satisfying affective and social integrative needs as well. In addition, they can help viewers relive stress and make them relaxed. Fourth, it is noteworthy that the frequency of consuming video game live stream content was positively associated with all the motivations in question. In view of the particular role of game-focused streams, namely a forerunner in the development of the global live stream industry, researchers have extensively investigated why people watch others play (Hamari and Sjöblom, 2017; Sjöblom and Hamari, 2017). The findings suggested that they usually do so for the purposes of learning gameplay knowledge and skills, seeking pleasure and enjoyment, integrating into the community, as well as escaping from daily routines (Sjöblom et al., 2017; Wulf et al., 2020; Vilasís-Pamos and Pires, 2021). Fifth, streams featuring food were closely aligned with the personal and social integrative motivations, in addition to the need for tension release. Such a content type can be epitomized by restaurant review, with contributors often being motivated by a sense of recognition in terms of their ratings of food, service, and atmosphere (Jalilvand et al., 2017; Hlee et al., 2019). There is also growing evidence demonstrating that Mukbang streaming can be conducive to alleviating stresses and tensions (Kircaburun et al., 2021; Liu and Kim, 2021). Sixth, although sports and fitness-related content was not necessarily associated with any of the psychological and social motivations of interest, education and cosmetics live streaming were found to meet the need for information seeking and knowledge acquisition (i.e., cognitive), both of which contain a considerable amount of instructional content. Finally, live stream viewers’ affective and personal integrative needs were gratified when they were watching cute pets. While the former is intuitive and deserves no further discussion, the latter might be rationalized by conceptualizing the pets, be they dogs or cats, as a projection of the viewer’s self (Veevers, 1985). People may achieve a sense of recognition if their virtual selves were approved or liked by others in the channels.

### Gratifications and support behaviors

According to the results shown in Table 4, the cognitive motivation had limited impacts on the support behaviors under consideration. In stark contrast, stress relief was found to be positively associated with all the support behaviors, which is in line with the extant literature surrounding live stream consumption (Todd and Melancon, 2017; Hilvert-Bruce et al., 2018; de Wit et al., 2020; Yoganathan et al., 2021). Aside from tension release, live stream viewers’ inclination for continuous watching was significantly affected by social integrative gratifications. In this sense, streaming channels can be likened to sports bars (Bulygin et al., 2020), where people gather and cheer for their favorite teams and players. The social integrative motivation also positively predicted the likelihoods of commenting and tipping. These two support behaviors embody a willingness to make proactive contributions to the community, differing primarily in whether monetary aid is involved. In regard to the remaining psychological and social gratifications, whereas the affective motivation merely increased viewers’ propensity to follow the channel, the personal integrative motivation encouraged all the support behaviors except continuous watching. This is the case probably because individuals who have a high level of self-confidence tend to participate in prosocial undertakings (McNeely and Meglino, 1994; Winterich et al., 2013).

**TABLE 4:** Direct effects of gratifications on support behaviors.

Motivation	Continue	Like	Follow	Comment	Tipping
Cognitive	–0.027	0.005	0.065	–0.039	–0.005
Affective	–0.054	0.060	0.121**	–0.048	–0.048
Personal integrative	0.019	0.062*	0.118***	0.233***	0.070**
Social integrative	0.271***	0.015	–0.030	0.249***	0.365***
Tension release	0.401***	0.437***	0.484***	0.272***	0.147*
*R* ^2^	0.487	0.386	0.502	0.465	0.352

*p < 0.05, **p < 0.01, ***p < 0.001.

### The mediating effects of gratifications

The estimated results of the mediation analysis appear in Table 5. In particular, to avoid lengthiness, we only report those whose indirect and total effects were both statistically significant. Among the five psychological and social motivations, it is worth noting that tension release functioned as a consistent mediator, fully or partially, for the relationship between content types and support behaviors. Although few prior studies have directly examined the mediating role of tension release within the context of live streaming, this result echoes the previous finding that different types of content vary in the efficacy in relieving stressful emotions (Sjöblom et al., 2017), which can subsequently influence users’ engagement in media consumption (Zolkepli and Kamarulzaman, 2015; Hamari and Sjöblom, 2017; Panda and Pandey, 2017). Quite a fraction of the remaining motivations played a mediating role as well. For example, the affective gratification mediated live stream content featuring cute pets and viewers’ tendency to follow, whereas watching food-themed streams indirectly increased the frequency of tapping the “like” button through the tension release mechanism.

**TABLE 5:** Direct, indirect, and total effects.

Path	Direct effect	Indirect effect	Total effect
E-commerce shopping → Affective → Follow	0.032	0.100***	0.132**
Beauty influencers → Affective → Follow	0.064	0.074**	0.138*
Entertaining plots → Affective → Follow	0.055	0.057**	0.112*
Video games → Affective → Follow	0.265***	0.102***	0.367***
Cute pets → Affective → Follow	0.115**	0.037*	0.152**
E-commerce shopping → Personal integrative → Like	0.138**	0.023*	0.161***
Video games → Personal integrative → Like	0.285***	0.037***	0.322***
Food → Personal integrative → Like	0.127**	0.026*	0.153***
E-commerce shopping → Personal integrative → Follow	0.165***	0.034*	0.199***
Video games →Personal integrative → Follow	0.317***	0.054***	0.371***
Food →Personal integrative → Follow	0.115**	0.038**	0.153**
Cute pets →Personal integrative → Follow	0.115**	0.051**	0.166***
E-commerce shopping → Personal integrative → Comment	0.063	0.051**	0.114**
Video games →Personal integrative → Comment	0.253***	0.082***	0.335***
Food →Personal integrative → Comment	0.156***	0.057**	0.213***
E-commerce shopping → Personal integrative → Tipping	0.184***	0.025*	0.209***
Video games →Personal integrative → Tipping	0.171***	0.039***	0.210***
Food →Personal integrative → Tipping	0.123**	0.028*	0.151***
Talent shows → Social integrative → Continue	–0.092	0.239***	0.147**
Video games →Social integrative → Continue	0.119***	0.208***	0.327***
Video games →Social integrative → Comment	0.147***	0.173***	0.320***
Beauty influencers → Social integrative → Tipping	0.038	0.093***	0.131*
Talent shows → Social integrative → Tipping	–0.082	0.191***	0.109*
Video games →Social integrative → Tipping	0.021	0.165***	0.186***
Talent shows → Tension release → Continue	–0.018	0.155***	0.137**
Video games → Tension release → Continue	0.108***	0.218***	0.326***
Talent shows → Tension release → Like	0.037	0.126***	0.163**
Video games → Tension release → Like	0.131***	0.178***	0.309***
Food → Tension release → Like	0.108*	0.078**	0.186***
Talent shows → Tension release → Follow	0.040	0.143***	0.183***
Video games → Tension release → Follow	0.169***	0.202***	0.371***
Talent shows → Tension release → Comment	–0.004	0.124***	0.120*
Video games → Tension release → Comment	0.145***	0.175***	0.320***
Food → Tension release → Comment	0.042	0.077**	0.119*
Talent shows → Tension release → Tipping	0.064	0.104***	0.168***
Video games → Tension release → Tipping	0.039	0.146***	0.185***

Considering the length of the estimation results, only those whose indirect and total effects were both statistically significant are reported here.

*p < 0.05, **p < 0.01, ***p < 0.001.

Content type-wise, video game live streaming was found to have indirect effects on all the five support behaviors under discussion, including continuous watching (*via* social integrative and tension release gratifications), like (*via* personal integrative and tension release gratifications), follow (*via* affective, personal integrative, and tension release gratifications), comment (*via* personal and social integrative and tension release gratifications), and tipping (*via* personal and social integrative and tension release gratifications). Having spearheaded the global fad of live stream services (Kaytoue et al., 2012; Recktenwald, 2017; Johnson and Woodcock, 2019), gaming-oriented content seems to continue to dominate the market whilst being effective in eliciting viewers’ support behaviors.

## Theoretical and practical implications

In the literature, limited attention has been given to the psychological and behavioral effects of different content types on live stream viewers, provided the rapid growth of the industry alongside greatly enriched content creation and provision. This topic is of considerable importance since the contemporary mix of media use can be characterized by the “attention economy” (Goldhaber, 1997; Davenport and Beck, 2001; Beller, 2012), wherein human attention is conceptualized as a scarce economic resource. That is to say, our cognitive capacity to process incoming messages is dwarfed by overflows of information, and hence in the realm of live streaming, we will not diversify indefinitely across content types, or even across channels within a given content type (Adler, 1985). In his seminal work, Simon (1955) coined the term “satisficing,” which is a hybrid of the two words “satisfy” and “suffice,” to describe individuals’ decision-making strategy, when coping with information overload, that pursues a locally satisfactory or adequate outcome rather than the globally optimal solution. As such, under the U&G framework, live stream viewers’ preferences for consuming different types of content should be closely aligned with how well their specific psychological and social needs are met. Our study makes important theoretical contributions to the communication literature by examining what kinds of gratifications can be satisfied by distinct live stream content types as well as what actions viewers would take to support their favored content types or channels as a result of psychological and social gratifications.

The findings obtained in this study also carry significant relevance for practitioners, such as content creators and live streamers. They can accentuate elements particularly attuned to increasing psychological and social gratifications, which in turn encourage desired support behaviors, depending on what type of content they specialize in. To be precise, for example, entertainment streams aimed at enlarging audience base might as well consider enriching affective communication, namely rendering the content more enjoyable, by incorporating violent humor (Blackford et al., 2011; Swani et al., 2013) or disparaging humor (Zillmann, 1983; Ford and Ferguson, 2004) to incentivize more viewers to follow. By the same token, video game streamers intending to monetize their audiences are suggested to broadcast playing casual games, such as *Stardew Valley* and the *Animal Crossing* series, with a relatable personality to help viewers relieve stress (Reinecke, 2009; Whitbourne et al., 2013), who then become more likely to send virtual gifts equivalent to financial rewards.

## Limitations and future research

This study faces several limitations that might restrain its contributions to the literature. The sample analyzed in this study was gleaned through an online survey, meaning that respondents were self-selected, who tended to be more active consumers of streaming service. Thus, the estimation results are prone to skewness toward the active population of live stream users. Moreover, content types were classified based on practical criteria as opposed to theoretical ones. Future research can try different methods to categorize the content types of live streaming, e.g., high- vs. low-level of novelty or humor use.

While the U&G framework should serve as an apt theoretical vehicle to delineate live stream viewers’ motivations related to distinct content types, the five psychological and social needs analyzed in this study might fail to sufficiently cover gratifications pertaining to viewership. For example, parasocial attachment can motivate media users to view specific contents and engage in particular support behaviors as well (Lim et al., 2020). Furthermore, although social interactions observed in this study were relevant mostly to the app’s digital society, which is governed by its unique rules and immaterial properties, previous research has demonstrated that digital societies’ values can be transferred to the real society and affect behaviors in the physical world. Wei and Gao (2017) found that the use of social media contributed to social integration of new urban migrants in China by helping them build social identity and social network, and also encouraged real-world social participation. By the same token, Hobbs et al.’s (2016) work suggested that online social integration is closely aligned with lower risk for a variety of critical health issues and therefore can be adopted to ameliorate modern populations’ social and physical health. What is more interesting is the pioneer work of Ahmedien (2020), who utilized convergence technologies to investigate the concept of “self-making” within the digital context, with a focus on generating lives based upon computer programs. Such innovations in bio-media technologies can help expand our understanding of biological and narrative identities of stem cells, even to the extent that identities can be controlled, enhanced, and resynthesized. Last but not least, our study only inspected live streaming on *Douyin*, which is extraordinarily prevailing in mainland China yet not the case in other parts of the world. Therefore, future research can consider undertaking cross-cultural examinations to see whether there exist cultural differences in preferences for content types, psychological and social gratifications, and support behaviors in relation to live stream consumption.

## Data availability statement

The raw data supporting the conclusions of this article will be made available by the authors, without undue reservation.

## Ethics statement

The studies involving human participants were reviewed and approved by the Ethics Committee of School of Cultural Creativity and Management, Communication University of Zhejiang. Written informed consent for participation was not required for this study in accordance with the national legislation and the institutional requirements.

## Author contributions

EM contributed to conception and design of the study, performed the statistical analysis, and wrote the first draft and sections of the manuscript.

## Footnotes

1
https://trendinsight.oceanengine.com

2
https://www.chanmama.com/douyin

3
https://www.feigua.cn/?chl=baidu-pinzhuan

4 Selected based on the numbers of viewers and total hours watched.
